# Serotonergic Medication Error: A Case Report of Serotonin Syndrome

**DOI:** 10.7759/cureus.48161

**Published:** 2023-11-02

**Authors:** Anh Thu N Nguyen, Riley G Fisher, Michael J Valentine, Ankur Kayastha, Hanh M Pham, Alexander T Phu, Saif A Meimon, Takara R Newsome-Cuby, Conor A Parry, Carol E Kirila

**Affiliations:** 1 College of Osteopathic Medicine, Kansas City University, Kansas City, USA; 2 College of Life Sciences, Brigham Young University, Provo, USA; 3 Public Health, University of Nebraska Medical Center, Omaha, USA

**Keywords:** serotonin-norepinephrine reuptake inhibitor, selective serotonin reuptake inhibitor (ssri), medical error, serotonin toxicity, atomoxetine, duloxetine, sertraline, mid-level care, parkinson disease, serotonin syndrome

## Abstract

Serotonin syndrome (SS) describes a life-threatening clinical condition that can develop within hours or days after taking serotonergic medication(s). Medication adverse reactions, overdose, or drug interactions can cause this syndrome. Patients often present with symptoms of hyperthermia, muscle rigidity, hyperreflexia, and clonus. Symptoms range broadly in severity, often influenced by polypharmacy and age. In this report, SS was diagnosed in an elderly patient who presented with diffuse urticaria and exacerbated tremor. These complaints were thought to be associated with Parkinson's disease due to a strong family history. Clinicians are encouraged to consider SS in their differential diagnosis when dealing with elderly patients with multiple medications, psychiatric diagnoses, conditions managed by other providers, and/or a strong family history of neurodegenerative diseases.

## Introduction

Serotonin syndrome (SS) manifests with diverse clinical presentations and potential for lethality. Various diagnostic criteria have been proposed, albeit the Hunter criteria are currently the most widely accepted. According to these criteria, a history of exposure to serotonergic drugs coupled with the presence of one or more symptoms such as hypertonia, agitation, tremor, hyperreflexia, elevated temperature, and clonus is indicative of SS [1]. Standardized protocols and confirmatory tests for SS are currently lacking. However, the general principles of management involve discontinuing the causative medication, providing supportive care, and administering benzodiazepines and/or cyproheptadine to mitigate symptoms associated with the syndrome. Notably, SS can manifest with varying severity across all age groups, ranging from subtle symptoms to a life-threatening condition [2]. Thus, a comprehensive understanding of this condition is imperative for all healthcare practitioners.

Given that diagnosis generally depends on clinical suspicion, SS has a low prevalence. Attar-Herzberg et al. have reported that a substantial number of SS cases were initially misdiagnosed as exacerbations of underlying psychiatric disorders before the correct diagnosis was established [3]. In one study of 112,045 hospitalized patients who were prescribed at least one serotonergic agent, six were diagnosed with SS. Upon closer inspection of medical records, they identified three cases that met full SS clinical criteria and 23 cases that met partial SS clinical criteria [4]. Further studies showed that the mortality rate is approximately 100 cases out of 7300 diagnosed cases of SS [4]. This underscores the significance of recognizing the elusive syndrome in primary care settings.

Parkinsonism is a term reserved for Parkinson's disease-like symptoms resulting from pharmacologic agents. Indeed, a more encompassing term commonly used is drug-induced parkinsonism. These symptoms include tremors, rigidity, and bradykinesia. In contrast, Parkinson's disease is a progressive neurodegenerative disease that manifests with similar extrapyramidal symptoms but without drug etiology. In both cases, dopamine blockade causes motor dysfunction, making it difficult for providers to distinguish the source. Notably, drug-induced parkinsonism is reported to be an underdiagnosed entity. It is most commonly associated with typical antipsychotics, atypical antipsychotics, and gastrokinetic agents [5]. 

Parkinson's disease is incurable. Instead, early management is focused on motor symptom amelioration. Dopamine agonists, catechol-O-methyl transferase inhibitors, and monoamine oxidase aldehyde dehydrogenase B (MAO-B) inhibitors are often used in an adjuvant treatment capacity. Levodopa is considered the first-line treatment as it has the most potent effect for therapy [5]. 

In this case report, we present the clinical scenario of a 79-year-old male with a past medical history of generalized anxiety disorder (GAD), post-traumatic stress disorder (PTSD), and a significant familial history of Parkinson's disease. This individual initially exhibited symptoms that did not readily align with SS. He presented with mild urticaria and agitation, which he attributed to seasonal allergies and PTSD. Due to this presumption and lack of physician-patient continuity, the patient was prescribed more than one serotonergic medication by multiple healthcare providers, one of whom was a nurse practitioner (NP). Due to the presence of bilateral diffuse urticaria and tremors, the patient was eventually diagnosed with SS. The aim of this case report is to increase recognition of SS and underscore risks associated with medication management errors. 

## Case presentation

A 79-year-old male presented to a family medicine outpatient clinic with diffuse urticaria and complaints of worsening tremor. He denied any known exposure to allergenic plants. Over the preceding week, these lesions had intensified. He reported distress due to the incessant need to scratch the affected areas. His past medical history was significant for GAD, PTSD, and a familial predisposition to Parkinson's disease. On prior consultations, he consistently reported escalating anxiety accompanied by observable mild tremors, which he postulated may be early signs of Parkinson's disease. Notably, his GAD was managed out of the office at a free clinic with an NP. 

Collected vital signs were as follows: a temperature of 99.1 degrees Fahrenheit, blood pressure reading of 110/78, heart rate of 85 beats per minute, and an oxygen saturation of 98% on room air. He weighed 240 pounds with a height of 69 inches (BMI = 35.4 kg/m^2^). On general examination, the patient appeared well-groomed. Dermatologic examination revealed diffuse hives on all limbs and excoriations from previous scratching. His posture was notably tense, as he was observed leaning forward in his seat with mild bilateral restless leg movements. Mild tremors that were nonspecific to resting, nor purposeful motor movement in the extremities, were observed throughout the interaction. Despite exhibiting a euthymic effect, he engaged in the discussion with an underlying nervous tone, presumably attributed to the discomfort from his dermatological symptoms compounded with GAD.

Informed consent was obtained from the patient for a steroid injection to treat his urticaria. Following this, a review of medications was performed. It was confirmed that this patient was prescribed a selective-serotonin reuptake inhibitor (SSRI) and two serotonin-norepinephrine reuptake inhibitors (SNRIs) from the NP to treat his mood disorder. Upon searching his recent pharmacy records, it was noted that he was simultaneously taking 50mg of sertraline, 50 mg of duloxetine, and 60 mg of atomoxetine. While it is appropriate to take an SSRI and SNRI, it is inappropriate to take multiple drugs of the same class. Taking duloxetine and atomoxetine concurrently is typically contraindicated. Upon further questioning, it became evident that the patient had been prescribed the additional SNRI (atomoxetine) by his nurse practitioner to manage his GAD.

It was estimated that the concomitant use of all three serotonin medications had been ongoing for three months. The patient was always careful about taking his medications correctly and properly managed his health. Through interviewing the patient, it was evaluated that he had no prior episodes of senile dementia and denied any possibility of misreading the prescription instructions. He admitted that he anticipated a follow-up call from the mid-level provider for further guidance after the atomoxetine prescription. However, this call never occurred. After an unsuccessful attempt to contact the clinic, he unilaterally decided to take all three medications. Thus, due to the confirmed intake of multiple serotonergic agents, a workup for serotonin syndrome was performed and he was promptly instructed to discontinue the atomoxetine. The exacerbation of this patient’s tremor, previously attributed to Parkinson familial history, may be sequela to serotonergic drug toxicity warranting SS workup.

Upon reflection, the patient determined it appropriate to cease GAD management from his mid-level provider. The patient was subsequently educated on signs of serotonin toxicity and he was ultimately referred to a neurologist for a comprehensive assessment. Further follow-up will be performed to discuss future psychiatric management.

## Discussion

**Figure 1:**
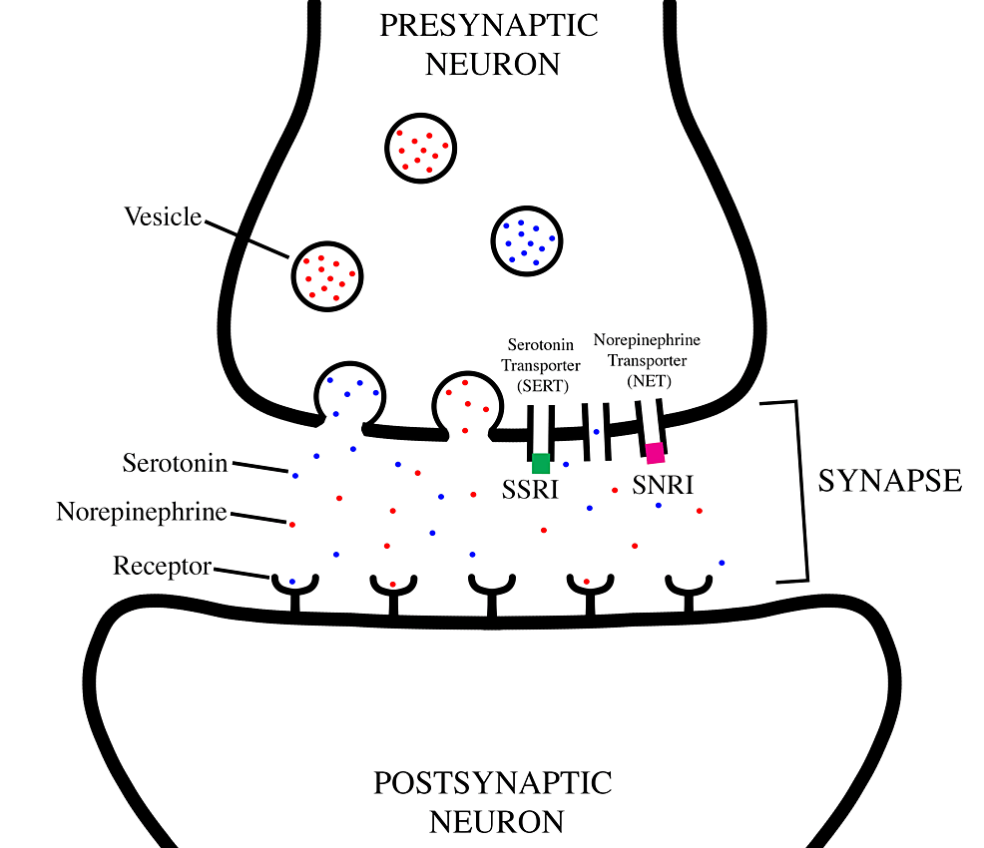
An illustration depicting the mechanism of action of selective-serotonin reuptake inhibitors and serotonin-norepinephrine reuptake inhibitors within a synaptic cleft. SSRI: Selective serotonin reuptake inhibitor, SNRI: serotonin-norepinephrine reuptake inhibitor, SERT: serotonin transporter, NET: norepinephrine transporter.

SS is a life-threatening condition characterized by an excessive accumulation of serotonin, often triggered by the administration of serotonergic drugs. SSRIs are the most frequently implicated. Monoamines (serotonin, dopamine, epinephrine, and norepinephrine) travel within the presynaptic neuron contained in vesicles. Once these vesicles reach the end of the presynaptic neuron, they release their contents into the synapse. These molecules may bind to their respective receptor located on the postsynaptic neuron or be taken up into the presynaptic neuron via their respective protein transporters in a process called reuptake. Selective serotonin reuptake inhibitors lead to an increased concentration of serotonin within the synaptic cleft. Similarly, serotonin-norepinephrine reuptake inhibitors operate by obstructing serotonin and norepinephrine reuptake, contributing to an elevated presence of both neurotransmitters within the synaptic cleft. An illustrative depiction providing an overview of the mechanisms associated with SSRIs and SNRIs can be found in Figure 1.

Serotonin and dopamine are members of the monoamine family and thus have similar molecular structures. While structure and function typically go hand in hand, the same cannot be said about these neurotransmitters. They are responsible for a plethora of physiologic mechanisms encompassing nearly all body systems. Generally speaking, dopamine is involved in physiologic processes related to movement, coordination, pleasure, and reward. Serotonin, on the other hand, is generally noted to be involved with emotional regulation and digestion. An in-depth explanation of all associated pathways and mechanisms is beyond the scope of this case report. However, it is noteworthy that both serotonin and dopamine imbalances can cause motor dysfunction. Dopamine may cause motor dysfunction with a deficiency of dopamine. Antipsychotic toxicity leading to dopamine blockade is a documented cause of extrapyramidal motor dysfunction. Contrastingly, serotonin may cause motor dysfunction in excessive concentrations, as seen in this case of observable tremors secondary to excessive serotonergic drug use. 

Both serotonin and dopamine are involved with life-threatening conditions called SS and neuroleptic malignant syndrome (NMS). While these conditions manifest with fever and autonomic instability, it is critical to differentiate each syndrome in clinical practice. SS is associated with clonus (hyperreflexia), whereas NMS is associated with rigidity. Furthermore, excessive serotonin will cause gastrointestinal upset. A summary of similarities and differences is found in Table 1.

**Table 1:** A summary of the similarities and differences of serotonin syndrome and neuroleptic malignant syndrome. This table does not contain an exhaustive list of symptoms (*). CK: Creatine kinase, WBCs: white blood cells

	Serotonin Syndrome (SS)*	Neuroleptic Malignant Syndrome (NMS)*
Etiology	Excessive serotonin	Deficient dopamine
Common symptoms	Fever and Autonomic Instability	Fever and Autonomic Instability
Distinguishing motor symptoms	Clonus (hyperreflexia)	Rigidity (muscular tone)
Other distinguishing factors	Gastrointestinal upset	Elevated CK and WBC

This case features an older male patient who developed an exacerbation of parkinsonism-like symptoms seemingly caused by SS after three months of exposure to multiple serotonergic drugs. Additionally, this patient had a strong family history of Parkinson's disease, making it difficult to determine the source of his motor dysfunction. It remains unclear whether this was secondary to serotonin toxicity, Parkinson's disease, or a combination of the two. While further workup is warranted to definitively exclude Parkinson's disease, there have been reported clinical cases with serotonin syndrome in the setting of Parkinson's disease [6,7].

This patient’s presentation satisfied both sets of Sternbach’s and Hunter's serotonin toxicity criteria via the clinical manifestation of anxiety, agitation, hypertonicity, rigidity, and tremors [4]. The severity of his SS was considered mild because he lacked severe features such as fever and autonomic instability. To date, there have been few reported cases of SS developing in prior diagnosed patients with Parkinson's disease. A past case report described SS in a patient taking MAO-B inhibitors while taking antidepressants intermittently [8]. Another case discussed a patient taking a combination of carbidopa-levodopa and sertraline, leading to SS [9]. There have been even fewer cases of reported SSRI-induced parkinsonism [9-11]. A case report from India by Dixit et al. described a case of SSRI-induced irreversible parkinsonism secondary to inhibitory effects of serotonin on dopamine neurotransmission, which may have altered functioning in the striatum [11]. 

While SS is a life-threatening condition, the severity is primarily based on clinical presentation. Sporadic clonus in the setting of febrile and hemodynamically unstable patients warrants a more rigorous management as compared to a patient presenting with mild symptoms. Thus, the management strategy of SS is largely supportive, focusing on discontinuation of the offending drug. In severe cases, typical management includes use of benzodiazepines for tremors, but antipsychotics are often avoided due to anticholinergic properties [2,12]. Management ultimately depends on clinical judgment. 

Other medical treatments are largely dependent on patient presentation, such as IV fluids, cooling measures for hyperthermia, and antihypertensives and/or vasopressors to treat autonomic instability. Even though cyproheptadine is a widely reported antidote for serotonin syndrome for its nonspecific serotonergic antagonistic properties, it remains primarily unproven [2]. For these reasons, treatment of SS continues to remain largely supportive. 

To our knowledge, there are few discussions in medical literature touching on polypharmacy from medical errors and overprescription of serotonergic drugs. Close management should be considered when including potential serotonin agents in treatment plans because they may induce dangerous side effects. While it cannot be ruled out that the patient experienced SS due to high doses of a combination of serotonergic medications, it is crucial to note that SS is more frequently observed in patients who are concurrently using such medication combinations [13]. In addition, even though elderly patients may have a history of senile dementia leading to an increased risk of difficult medicinal management, this was not the case in our case presentation [14]. 

It is crucial for practicing clinicians to consider SS as one of the potential adverse consequences when prescribing elderly patients multiple serotonergic drugs. The diagnosis of SS is largely considered a diagnosis of exclusion, requiring the clinician to have experience and knowledge when managing patients with polypharmacy. In particular, the prescribing clinician should utilize prudent monitoring for therapeutic benefits prior to starting additional serotonergic drugs. A target goal is to establish maintenance dosage for at least six months [15,16]. Previous guideline recommendations allowed prescription of multiple serotonergic drugs with different mechanisms of action to optimize treatment effect. However, this approach is not currently recommended in practice guidelines of most countries, let alone prescribing multiple medications within the same class [15,16]. Therefore, it is of utmost importance to educate all levels of prescribing clinicians, from physicians to mid-levels, to prescribe serotonergic drugs with caution in order to reduce prescription errors and potential serotonergic toxicity, leading to clinical manifestations of serotonin syndrome.

While it is important to conduct root-cause analysis and prevent medication errors, several factors must be taken into consideration prior to developing conclusions. Firstly, the mid-level practitioner operating in a free clinic may not have access to the same electronic medical records (EMRs). While the NP should have patient medical records, different EMRs are notorious for being difficult to use, therefore confusion may arise when interpreting these records by not just NPs, but any practicing clinician. While the patient denied recall of prior episodes of dementia or misunderstanding medication instructions, this must still be taken into consideration given the patient’s age. Therefore, the need for clear language on patient medication instructions and employment of the teach-back system with patients is crucial. In addition, this error reflects potential multi-level errors that are much broader than medication error by one clinician. Pharmacies should also be error-checking when multiple concomitant drugs of the same class are prescribed. Serotonin syndrome can have dangerous implications, leading to severe consequences and potential lethality. All levels of prescribing clinicians should openly discuss and accept responsibility for side effects resulting from polypharmacy and medical errors. It is of utmost importance to consider family history of neurodegenerative conditions to continually improve patient safety.

## Conclusions

The incidence of SS may be infrequent, but its potentially life-threatening nature requires careful investigation. The most common causes include intentional overdose of serotonergic medications (monotherapy) or exposure to multiple serotonin agents (polypharmacy). This case study demonstrates that patient-clinician discontinuity is an important factor that increases medical errors associated with overprescribing. In addition, this case highlights the crucial need to have EMRs and medical records as easy to operate and interpret as possible to prevent any confusion or missing patient information. It is also crucial that each member of the healthcare system works as a broader team in order to prevent clinical mistakes. 

Clinical features of serotonin toxicity typically manifest as a constellation of symptoms, including but not limited to fever, autonomic dysregulation, clonus, tremor, and heightened deep tendon reflexes. While the clinical picture may bear resemblance to NMS, differentiation between the two syndromes can often be achieved through a rigorous review of the patient's pharmacological history and the distinguishing symptom of clonus as the primary motor dysfunction in SS. Furthermore, the source of motor dysfunction may be difficult to discern in the setting of Parkinson's disease and other etiologies of parkinsonism. Given the lethal implications of SS, all levels of practicing clinicians are advised to exercise caution when subjecting patients to pharmacological agents with serotonergic properties. It is imperative that all members of the healthcare team prioritize patient safety as the paramount objective in their therapeutic management strategies.
